# Joint Task Force for Clinical Trial Competency and Clinical Research Professional Workforce Development

**DOI:** 10.3389/fphar.2018.01148

**Published:** 2018-10-16

**Authors:** Stephen A. Sonstein, Carolynn T. Jones

**Affiliations:** ^1^Multiregional Clinical Trial Center, Brigham and Women’s Hospital and Harvard, Cambridge, MA, United States; ^2^College of Nursing, The Ohio State University, Columbus, OH, United States

**Keywords:** competence, competency, workforce development, clinical research, portfolio, professional development, accreditation

## Abstract

Clinical research workforce development efforts have focused on both increasing the size of the workforce of investigators and professionals working in the clinical research enterprise, but also the education and training of those individuals to ensure the quality of study performance to improve the public’s health. A major contribution to these efforts has been the establishment of core competencies for clinical research professionals by the Joint Task Force for Clinical Trial Competency. This article reviews the development of the clinical research core competencies, their wide adoption and influence on job descriptions, education, training, and academic accreditation.

## Introduction

It is widely agreed upon that there has been a significant increase in both the number and the complexity of clinical trials during the past decade. The number of registered clinical trials as of June 22, 2018 is 276,190 up from 231,208 just a year ago ^1^. The global clinical trial service market is predicted to reach $64B by 2020 (Centerwatch, 2017). The demand for clinical research professionals (CRPs) already exceeds the supply and the pressure to grow the clinical research workforce will undoubtedly continue. The underlying solution is much more complex than just recruiting, educating and training new students to become CRPs and increase the size of the workforce.

## Developing the Clinical Research Workforce

There is no required educational background or defined set of competencies that are necessary to become a CRP. The majority of the current workforce has been trained “on the job.” Very few enter the clinical research profession as a direct result of undergraduate education or knowledge of the field. An understanding of the professional roles in clinical research, adequate onboarding, an understanding of the ethical underpinnings of the profession and an ability to grow professionally and move upward in an organization are key elements that are required to be successful in the field. Onboarding training in clinical research is typically minimal or poorly organized (Sonstein et al., 2014). Though most individuals, with time, become skilled at their current roles, as the responsibilities of their role changes with increasing technological and quality demands, or new opportunities arise, individuals find themselves moving from proficient to novice repeatedly. In this era of increasing role complexity, the lack of professional requirements and potential educational gaps can lead to role dissatisfaction and personnel turn-over, a costly by-product.

During the past decade academic programs have been developed to educate and train physician investigators, clinical research coordinators, clinical trial monitors, regulatory affairs professionals, and clinical data managers. The graduates from these programs are highly qualified and anxious to enter the market, but unfortunately, the hiring criteria almost always require varying levels of previous experience. It is assumed that experience equates with competence. For most other health-related professions, professional certification or licensure is recognized as competence. Entry level individuals are required to have a specific academic degree, often an internship or other hands-on experience and have passed an examination which is administered under the aegis of a representative professional or licensure organization.

There are two widely recognized professional organizations which offer professional certification to CRPs. The ACRPs and Society for Clinical Research Associates (SoCRA) both require a minimum of 2 years’ documented clinical research work experience to be eligible for their qualifying examinations (Association of Clinical Research Professionals, 2018b; Society of Clinical Research Associates, 2018). The 2-year standard, based solely on time of employment rather than competence, contributes to the current shortage of clinical research workforce personnel. Individuals who have completed academic programs in clinical research are still required to document previous experience in order to qualify to sit for certification examinations, but the requirement may be lessened to 1 year for approved programs. Nevertheless, this experience criterion has created a “Catch-22” situation where you need experience to get a job and professional certification, but you need a job to get experience and professional certification.

Not only does the clinical research workforce solution require an influx of new qualified professionals, but it requires that the current workforce continuously enhance their competency through professional development activities. Clinical research quality assurance and training requirements are mandated in the updated International Council for Harmonization of Technical Requirements for Pharmaceuticals for Human Use (ICH) Guidelines (International Council for Hamronisation of Technical Requirements for Pharmaceuticals in Human Use, 2016) and the Declaration of Helsinki (World Medical Association, 2013), but again, there has been until recently, no generally agreed upon set of core competencies upon which educational programs and training requirements for either entry level professionals or continuing professional development would be based.

## The Joint Task Force for Clinical Trial Competency- Setting the Professional Standard

Professionalization is defined as the process by which “any trade or occupation transforms itself through the development of formal qualification based upon education, apprenticeship, and examinations, the emergence of regulatory bodies with powers to admit and discipline members, and some degree of monopoly rights” (Bullock and Trombley, 1999). Roles associated with CRPs have evolved through a process of delegation from principal investigators (Mueller, 2001). Over time, those roles have evolved and resulted in a workforce of highly skilled professionals that are integrated across the clinical research enterprise. To become recognized as a profession, certain characteristics should be in place. Hinkley et al. (2015) highlight these characteristics as a “mature” profession being: (1) accredited professional education; (2) skills development (core competencies); (3), Licensing; (4) Professional Development; and (5) Code of Ethics. Licensing of all members of the clinical research profession is a controversial issue, but may be a necessary characteristic depending on role and scope of practice (e.g., clinical research nurse; physician principal investigator, pharmacist, etc.). Overall, the still evolving profession of clinical research is meeting these characteristics and continues to make significant strides toward maturing the profession and in workforce development.

**FIGURE 1:**
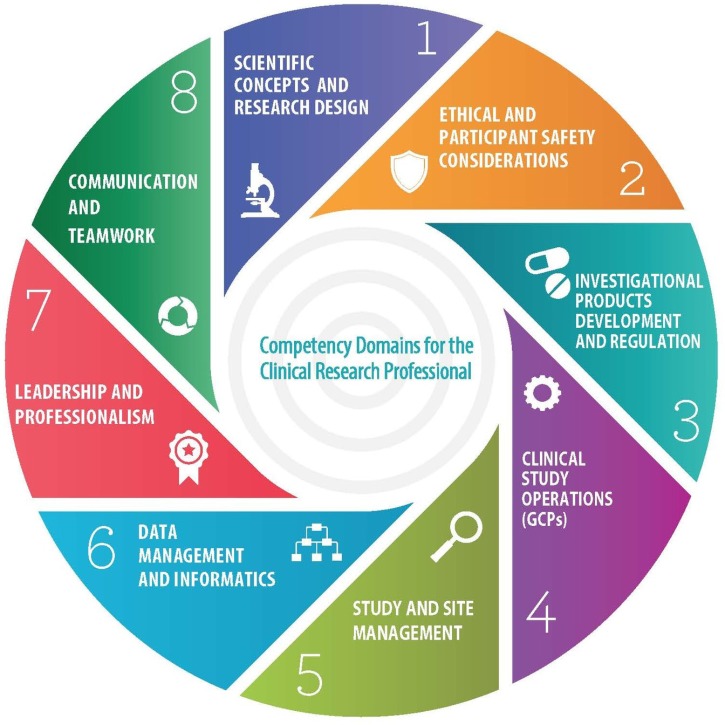
Joint Task Force for Clinical Trial Competency Core Competency Domains.

During the spring of 2013, at a meeting of representatives from pharmaceutical companies, contract research organizations, academic institutions, clinical research sites and professional societies, hosted by the MRCT at Harvard University, the JTF was formed. During the following year, the JTF members collaborated to develop a single, high-level set of standards that could be adopted globally and serve as a framework for defining professional competence throughout the clinical research enterprise. The JTF Framework is composed of 8 Domains (**Figure 1**) and 48 core competency statements which were aligned and harmonized utilizing published statements of core competency requirements which cover the entire clinical research enterprise.

The standards developed by the JTF incorporated formal input from a variety of United States and international stakeholders from academic institutions, non-profit organizations and the private sector (Sonstein et al., 2014). The JTF Core Competency Framework has been widely recognized globally as the standard for skills development and competency (Kremidas, 2017). Many federally-sponsored research initiatives in the United States have adopted the JTF Framework to help define workforce development at Clinical and Translational Science Award funded institutions (Calvin-Naylor et al., 2017). Materials and adoptions of the JTF Framework have been widely disseminated at conferences, in the manuscripts cited herein, and on the JTF Website (Joint Task Force for Clinical Trial Competency, 2017b). The JTF is currently receiving administrative and website support from the Multi-Regional Clinical Trials Center of Brigham and Women’s Hospital and Harvard University. One of the highly significant adopters of the JTF Framework has been by ACRP. This global organization represents more than 13,000 CRPs and has restructured their annual meeting into sessions which follow the JTF Framework Domains. As concerns professional certification, ACRP has realigned their personnel certification examinations based upon the same Domains. Following suit, the SoCRA also re-aligned their certification exam to the JTF Framework. While licensing is not required for all roles in the clinical research profession, it is applicable for specific clinical roles. Moreover, certification has become a standard in the profession.

The JTF acknowledged that the dynamic nature of the clinical research enterprise would necessitate that the JTF Framework would require ongoing updating as technological and regulatory changes occurred to the clinical trial process. Recently, the JTF Framework was updated to clarify terminology, to refine the organization and description of certain competencies, and to be inclusive of clinical research beyond clinical trials alone (Joint Task Force for Clinical Trial Competency, 2017a). This can be viewed online at the JTF Website. Ongoing review by the JTF will determine future updates of the Framework.

Traditionally, the academic recognition of a profession is made by peers and by organized practitioners through an accrediting body. In 2012, the profession of clinical research was recognized by the CAAHEPs, opening the door to the establishment of curriculum standards and a pathway to clinical research education program accreditation by CAAHEP (Commission on Accreditation of Allied Health Programs, 2012). There are currently 100s of academic programs which educate CRPs in United States, Canada, Europe, Africa, Asia, India, and Australia. Many of these programs are members of the COAPCR and have mapped their curricula to the JTF Core Competencies in preparation for future accreditation applications. Checklists to aid in training or academic course mapping can be found on the JTF website http://clinicaltrialcompetency.org.

Further compromising clinical research workforce development has been inconsistency in job titles and professional progression. A recent survey of CRPs indicates that the most common reason for turnover among CRPs is lack of professional progression, training opportunities, and professional development (Applied Clinical Trials, 2018). The JTF Framework has been used to address job predictability and professional advancement. Duke University utilized the JTF Framework to restructure job titles and progression pathways which reduced the number of job titles from 80 to 12 and led to greater consistency and predictability (Brouwer et al., 2017b). Professional descriptions further defined levels of clinical research experience as Fundamental, Skilled and Advanced in order to create professional ladders. Others have also utilized the JTF Framework to define specific role responsibilities (Association of Clinical Research Professionals, 2017, 2018a). In 2016, the American Nurses Association recognized the specialty of Clinical Research Nursing (American Nurses Association and International Association of Clinical Research Nurses, 2016).

Members of the JTF have participated in numerous international collaborations which are highlighted on the JTF Website ^2^. For instance, PRAXIS Australia, Ltd. created Research Essentials comprised of 68 accessible modules and electives which is based on the JTF Framework and the United Kingdom NIHR Clinical Research Network has used a competency-based approach to create the Integrated Workforce Framework (Joint Task Force, 2018). International training efforts are being developed by JTF members to include competency-based modules for workforce development in India and in South America (personal communication, Jones, 2018) and via PharmaTrain.

Ongoing debate related to education, experience, and hiring practices prompted a global survey of CRPs that addressed self-perceived competence and the relevance of the JTF Framework to their roles (Sonstein et al., 2016). Responses to the survey were received from CRPs in the United States, Europe, Latin America, Asia, and Australia and represented all of the major professional roles. Analysis of the results of the survey showed that the domains and core competency statements within the JTF Framework were relevant globally, but also indicated that specific roles differed between regions in their competency requirements. Additionally, it became clear that there was a need to acknowledge the increase in level of competence that occurs as individuals move forward in their careers. As one gains experience and moves into a leadership or mentoring role, the level of competence should increase. In addition, certain roles within the enterprise require differing levels of competence in different domains. For example, a study site supervisor in a data management role would need high level competencies in the Data Management and Informatics and the Leadership and Professionalism Domains, but would not require such competencies in the Scientific Concepts and Research Design or Investigational Products Development and Regulation domains. As noted above, it has been shown that in different areas of the world and under different regulatory authorities, the competency requirements for certain roles differ. For many South American countries, for example, the role of Clinical Research Coordinator is uncommon: Principal Investigators (PIs) are directly responsible for clinical trial implementation (Silva et al., 2017). Thus, PIs in this region of the world would need higher level competencies in the Clinical Trial Operations and Site Management Domains.

## Applying a Leveled Approach for Targeted Workforce Development

Clinical research stakeholders suggested that broader adoption and utility of the JTF Framework would be facilitated by defining the competencies at Fundamental, Skilled and Advanced levels so that they could be applied across a wider range of roles. A recent manuscript reviews the process undertaken by the JTF to generate leveled competencies for CRPs across the broad spectrum of roles that characterize the enterprise and the product of that effort (Sonstein et al., in press). Examples of measurable competency assessments at each level are provided as a supplement to this manuscript and are designed to facilitate their application in workforce development initiatives across the clinical research enterprise.

Several groups have targeted a similar approach for clinical research competencies or skillsets for particular roles in clinical research. The ACRP has used a stakeholder approach to develop leveled competencies for study coordinators and clinical research monitors, two groups that ACRP has targeted for certification (Association of Clinical Research Professionals, 2017, 2018a). The Oncology Nursing Society published the 2016 Oncology Clinical Trials Nurse Competencies, updating their previous versions to include a leveling approach (Oncology Nursing Society, 2016). The United Kingdom NIHR produced an Integrated Workforce Framework as a resource for CRPs and nurses working in the NIHR Clinical Research Network that is intended to be used as a self-assessment tool for four levels of CRPs (National Institute for Health Research, 2017). The Regulatory Affairs Professional Society has produced a leveled approach for their Regulatory Affairs Core Competencies (Regulatory Affairs Professional Society, 2016). The Global Health Network has launched a competency framework for low and middle income global clinical researchers working in tropical diseases (Training in Tropical Diseases and The Global Health Network, 2016). Duke University applied the JTF Framework to employ a “tiered” approach to professional progression across several job families, such as study coordinators, clinical research nurses, regulatory coordinators, and research program managers (Brouwer et al., 2017a,b).

Intentionally, the JTF leveling work was not directed to any specific role within the clinical research enterprise. In addition, the international relevance of the effort was ensured by including non-United States representatives in each of the five workgroups which contributed to the work. The JTF Leveled Competency Framework (see **Supplementary Table 1**) is intended to provide direction for those who are creating training programs, specialized role descriptions, or professional progression planning for clinical research positions and may be adjusted to site-specific practice cultures. This framework helps to define the central skills and competencies that professionals at various levels of proficiency require in order to plan, conduct, or manage clinical research. Moreover, this leveled competency framework is intended to be internationally applicable.

One of the more difficult aspects of workforce development is the actual assessment of an individual’s competence. The JTF used Bloom’s Modified Taxonomy (Anderson and Krathwohl, 2001) and developed specific examples for each competency level which provided an intentional approach that described KSAs, ranging from “novice to expert” (Benner, 1984), as an initial step to use for competency assessment and consistent expectations in an organization, particularly when it is difficult to align across departments, divisions, research groups, or global regions.

The clinical research enterprise employs individuals in a variety of roles and at varying levels of expertise. Given the global expansion and increasing complexity of the enterprise, and the documented shortage of qualified clinical research personnel, addressing the workforce development needs has become a priority worldwide (Li et al., 2015; Miseta, 2016; Howes, 2017; The Economist Intelligence Unit, 2018). The JTF Framework Fundamental level provides a series of standards to describe the role and training needs of new entry level professionals. The Skilled level competencies define the professional expectations for experienced, mid-career CRPs and can serve as a model for upward mobility and career development. The Advanced level competencies provide guidelines for managers, mentors and other leadership roles within the enterprise as well as aspirational goals for those hoping to move into leadership roles.

The Leveled JTF Framework complements previous work and offers a generic entry level goal for the education and training of new CRPs; a definition of demonstrated competencies that human resource professionals, managers, and educators can use for assessments and career mobility; and goals for leadership and mentoring roles within the clinical research enterprise. The provision of the additional granularity of “levels” to the individual competencies, and the assessment criteria, renders transparent a pathway for professional development and organizational consistency. Using a leveled approach to writing job descriptions or progression planning, individuals have a more objective method of professional role progression which leads to better staff opportunity and job satisfaction.

## Continuing Professional Development and Competency Assessment

As more groups adopt the JTF Framework, new approaches for assessment of competence, the application of individual professional development planning (IDP) and ePortfolios may stimulate workforce development and demonstrated competence. Portfolios are collections of educational and work-related documents that showcase the progression of acquired KSAs as a learner or professional. Early use of portfolios was paper-based, where documents were collected and filed in a notebook. Electronic formats for portfolios, called ePortfolios are now being used. Not only are portfolios used to showcase work, they can also be used to document continuing education and professional activities and as a forum for documenting development goals. EPortfolios are used as an assessment tool for professional role development. Moreover, the use of ILPs or “individualized learning plans” have been used in medical education whereby medical residents engage in competency-based self-directed learning by setting short-term SMART (specific, measurable, accountable, realistic, timely) goals and a mentored approach for demonstrating goal and learning acquisition (Hernandez et al., 2017; Kastenmeier et al., 2018). Similarly, IDPs have been used in mentored clinical and translational investigator training to expand the numbers of clinician-scientists (Fuhrmann et al., 2018). The JTF Framework has been used as a competency-based approach to stimulate individual professional and learning development and to demonstrate KSAs through ePortfolios, in formal education and workforce development areas (Association of Clinical Research Professionals, 2016; Jones et al., 2016; The Ohio State University, 2018). Such applications of ePortfolio are being implemented in CRP development across federally-funded research sites in the United States; as a potential system to improve the assessment of applicants to contract research organization jobs (personal communication, W. Gluck); and, to assist in measuring acquired KSAs for job promotions (Shiner, 2009).

Elements of an ePortfolio system would include showcasing KSAs that illustrate competence by JTF Core Competency Domain (**Figure 1**). For example, a study coordinator may upload an SOP, a recruitment plan, a participant educational flier, and an innovative informed consent checklist to an ePortfolio to show “experience” in the Ethical and Participant Safety and Clinical Trials Operations domains. The ePortfolio model provides a competency-based approach for showcasing experience and can be used for internal organizational evaluation and upward mobility as well as by an individual seeking employment to demonstrate competence and supplement existing social media sites such as LinkedIn. Today’s employers are more influenced by an ePortfolio over a paper-based resume (Leahy and Filiatrault, 2017).

## Conclusion

In Summary, the JTF Framework has become a global resource. Though regulatory guidelines and implementation mechanisms differ from region to region, the JTF Framework provides a universal standard and a valuable foundation for initiatives that are seeking to increase the size, competency, and professionalization of the workforce responsible for the design, conduct, and oversight of clinical research.

## Author Contributions

SS and CJ contributed equally to the writing and editing of this manuscript.

## Conflict of Interest Statement

The authors declare that the research was conducted in the absence of any commercial or financial relationships that could be construed as a potential conflict of interest. The handling Editor and two reviewers PS and SK-F declared their involvement as co-editors in the Research Topic.

## Footnotes

1 ClinicalTrials.gov

2 www.clinicaltrialcompetency.org

## Abbreviations

ACRPs: Association of Clinical Research Professionals
CAAHEPs: Commission on Accreditation of Allied Health Education Programs
CoAPCR: Consortium of Academic Programs in Clinical Research
CRP: clinical research professional
ICH: International Council on Harmonisation of Technical Requirements for Pharmaceuticals for Human Use
IDP: individual development plan
JTF: Joint Task Force for Clinical Trial Competency
KSAs: knowledge, skills and attitudes
MRCT: Multi-Regional Clinical Trial Center
NIHR: National Institute for Health Research
SoCRA: Society of Clinical Research Associates
SOP: standard operating procedure
