# Supplementary material for: Joint Task Force for Clinical Trial Competency and Clinical Research Professional Workforce Development
Source: Front Pharmacol. 2018 Oct 16;9:1148. doi: 10.3389/fphar.2018.01148 (PMC6198073; doi:10.3389/fphar.2018.01148)
Supplement: Supplementary file 1 [file Table_1.DOCX]

| **Core Competency Framework for the Clinical Research Professional, Version 2.0**  **FUNDAMENTAL, SKILLED and ADVANCED LEVEL** | | | | | | | |
| --- | --- | --- | --- | --- | --- | --- | --- |
| **DOMAIN 1: Scientific Concepts and Research Design:** Encompasses knowledge of scientific concepts related to the design and analysis of clinical trials | | | | | | | |
| 1.1 | Apply principles of biomedical science to investigational product discovery and development and health-related behavioral interventions | | | | | | |
|  | **Fundamental Level**  Researcher can:   1. **Recognize** the need to apply scientific principles to discovery and development of biomedical investigational products and health-related behavioral interventions 2. **Explain** the basic scientific principles that should be applied during development of biomedical investigational products and health-related behavioral interventions   **Example:** *When reviewing a clinical research protocol, researcher describes the objective and scientific techniques used to design and implement biomedical research.* | | | **Skilled Level**  Researcher meets the Fundamental Level AND can:   1. **Apply** scientific principles when implementing a clinical or behavioral study 2. **Implement** data collection according to scientific principles and based on protocol design   **Example**: *When given a clinical research protocol, researcher differentiates* *what principles could affect how the data should be collected and implement best practices accordingly.* | | | **Advanced Level**  Researcher meets the Skilled Level AND can:   1. **Plan** biomedical research according to scientific principles 2. **Develop** a data management plan according to scientific principles   **Example**: *Given a clinical research protocol and data collected, the researcher evaluates the findings to assess results via a scientific framework.* |
| 1.2 | Identify scientific questions that are potentially testable clinical research hypotheses | | | | | | |
|  | **Fundamental Level**  Researcher can:   1. **Articulate** the purpose of the study 2. **Describe** the importance of the study   **Example:** *Identifies the following elements in selected study protocols: Study title, Key purpose of the study, Why this study is important to be done, Who the specific population for the study is* | | **Skilled Level**  Researcher meets the Fundamental Level AND can:   1. **Identify** the research hypothesis in a study protocol 2. **Identify** endpoints (primary and secondary) that will be used in data analyses to measure outcomes   **Example:** *When given a study protocol, describes and classifies the objectives and associated safety and efficacy endpoints that will be used to test the hypothesis and identify assessments (clinical, social/ behavioral, or economic) that will be used to measure endpoints.* | | **Advanced Level**  Researcher meets the Skilled Level AND can:   1. **Develop** protocol or source document checklist language that identifies the scientific questions (hypotheses), primary objectives, secondary objectives, and associated endpoints 2. **Align** parameters for collecting data on endpoints with objectives   **Example:** *Develops presentations to educate others on the scientific feasibility and conduct of the study to ensure quality collection of endpoints for hypothesis testing.* | | |
| 1.3 | Identify the elements and explain the principles and processes of designing a clinical study | | | | | | |
|  | **Fundamental Level**  Researcher can:   1. **Identify** the key elements of a clinical study protocol 2. **Describe** the general process of clinical study protocol development 3. **Recognize** the basic differences between the various types of clinical studies   **Example:** *When given a clinical study protocol, identifies the inclusion and exclusion criteria for a set of mock participants.* | | **Skilled Level**  Researcher meets the Fundamental Level AND can:   1. R**eview** a clinical study protocol to ensure all needed elements are included   **Example:** *When given a clinical study protocol, identifies missing, incomplete or inappropriate features.* | | **Advanced Level**  Researcher meets the Skilled Level AND can:   1. **Evaluate** the clinical study design and **make adjustments** to the processes as needed 2. **Develop** protocols as applicable to the therapeutic area 3. **Evaluate** strengths and weakness of study designs and explain these to others   **Example:** *When given a clinical study protocol that has misalignment between the measures and objectives, researcher appropriately modifies the protocol.* | | |
| 1.4 | Critically analyze clinical study results | | | | | | |
|  | **Fundamental Level**  Researcher can:   1. **Identify** the study results 2. **Describe** the relevance of the results to the research question   **Example:** *When given study reports, paraphrases and summarizes the study results.* | | **Skilled Level**  Researcher meets the Fundamental Level AND can:   1. **Compare and assess** the level of quality of results associated with study reports and publications 2. **Understand** descriptive and exploratory data analysis   **Example:** *When given two publications researching the same topic, researcher compares and contrasts what could have affected how the data from the two could be interpreted.* | | **Advanced Level**  Researcher meets the Skilled Level AND can:   1. **Assess** the potential for application of findings 2. **Identify** trends and anomalies within the clinical study data   **Example:** *Conducts pharmacovigilance assessments of collected data and generates queries to close data gaps* | | |
| **DOMAIN 2: Ethical and Participant Safety Considerations:** *Encompasses care of patients, aspects of human subject protection, and safety in the conduct of a clinical trial* | | | | | | | |
| 2.1 | Differentiate between standard of care and clinical study activities | | | | | | |
|  | **Fundamental Level**  Researcher can:   1. **Explain** that a clinical study is unconfirmed research and not accepted standard of care   **Example:** *Explains to a study participant that procedures that are part of the protocol are not necessarily standard of care.* | | **Skilled Level**  Researcher meets the Fundamental Level AND can:   1. **Demonstrate** the importance of conducting clinical trial activities as per the protocol   **Example:** *Explains to clinical staff the timing of a research blood draw versus standard blood draw timing for the shift.* | | **Advanced Level**  Researcher meets the Skilled Level AND can:   1. **Develop** a protocol that appropriately includes distinct research activities and standard of care   **Example:** *Appropriately distinguishes between activities that should be billed to insurance versus incorporated into sponsored cost.* | | |
| 2.2 | Define the concepts of “clinical equipoise” and “therapeutic misconception” as they relate to the conduct of a clinical study | | | | | | |
|  | **Fundamental Level**  Researcher can:   1. **Recognize** that clinical equipoise and therapeutic misconception are fundamental ethical principles and concerns that underlie clinical research   **Example:** *Identifies and discusses the two comparators in a controlled clinical trial and why each has been selected.* | | **Skilled Level**  Researcher meets the Fundamental Level AND can:   1. **Explain** the rationale of clinical equipoise and therapeutic misconception, and can demonstrate comprehensive knowledge and understanding of how they may impact patient understanding 2. Consistently **apply** knowledge of clinical equipoise and therapeutic misconception during the course of the study 3. **Recognize, interpret,** and seek assistance where required to address participant concerns regarding therapeutic misconception or clinical equipoise   **Example:** *Identifies during ICF process whether the potential participant truly understands the study is research and does not have a predictable outcome.* | | **Advanced Level**  Researcher meets the Skilled Level AND can:   1. **Act** as an expert resource to potential study participants and staff in their understanding of clinical equipoise and therapeutic misconception   **Example:** *Leads the development of an in-service training by interpreting study protocols in relation to clinical equipoise and therapeutic misconception.* | | |
| 2.3 | Apply relevant national and international principles of human subject protections and privacy throughout all stages of a clinical study | | | | | | |
|  | **Fundamental Level**  Researcher can:   1. **Explain** the importance of complying with global guidelines and recommendations, as well as local regulations regarding the safety, wellbeing, and rights of all subjects participating in a clinical trial anywhere   **Example:** *Identifies examples of autonomy, justice and beneficence in the recruitment and consent process for a clinical protocol* | | **Skilled Level**  Researcher meets the Fundamental Level AND can:   1. Critically **appraise and implement** within a clinical study protocol, the principles of human subject protection and privacy   **Example:** *Designs recruitment strategies that ensures inclusion of all appropriate populations.* | | **Advanced Level**  Researcher meets the Skilled Level AND can:   1. **Supervise** the implementation of activities required to protect a clinical study participant´s privacy, safety, wellbeing, and rights in a clinical trial being conducted in any region 2. **Respond** to questions posed by a regulatory body (e.g. IRB.IEC) regarding the methods by which a clinical study protects the privacy and safety of participants   **Example:** *Explains to an IRB/IEC the plans for ensuring participant confidentiality for a clinical study being submitted for review.* | | |
| 2.4 | Explain the evolution of the requirement for informed consent from research participants and the principles and content of the key documents that ensure the protection of human participants in clinical research | | | | | | |
|  | **Fundamental Level**  Researcher can:   1. **Identify** the historical events which have led to the development of the current informed consent regulations 2. **Identify** the key documents that ensure the protection of human participants in clinical research (Declaration of Helsinki, Belmont Report, CIOMS, Nuremberg report, ICH guidelines, Investigators Brochure, product label, etc.)   **Example:** *Identifies and explains the three principles of the Belmont Report and the difference between FDA regulations and ICH GCP guidelines.* | | **Skilled Level**  Researcher meets the Fundamental Level AND can:   1. **Recognize** the critical nature of communicating the potential risks or hazards, as well as the benefits of a clinical study, using terminology and a manner that is understandable by the potential study participants during the informed consent process 2. **Apply** knowledge of the key doctrines and tenants for the regulations and guidelines coupled with available safety information when drafting an informed consent document for a clinical study   **Example:** *Composes the informed consent document for a clinical study and includes the potential risks and benefits in an understandable manner for the study participants.* | | **Advanced Level**  Researcher meets the Skilled Level AND can:   1. **Implement** processes and control measures to ensure human subject protection regulations requirements are met across studies 2. **Evaluate** the informed consent document in relationship to the study protocol to assure that it not only meets current regulations and guidelines but also provides the information needed for a potential study participant to make an informed decision regarding their participation in the study   **Example:** *Serves as an effective member of an IRB to ensure human subject protection.* | | |
| 2.5 | Describe the ethical issues involved when dealing with vulnerable populations and what additional safeguards should be in place for those populations | | | | | | |
|  | **Fundamental Level**  Researcher can:   1. **Identify** which populations are considered vulnerable 2. **Understand** that regulations are in place to protect vulnerable populations   **Example:** *Understands these groups as being vulnerable: children, prisoners, pregnant women, mentally disabled persons, and economically or educationally disadvantaged persons and accurately describe additional safeguards in place for each group.* | | **Skilled Level**  Researcher meets the Fundamental Level AND can:   1. Accurately **apply** the appropriate safeguards with research participants 2. **Anticipate** situations when research participants may be considered vulnerable   **Example:** *Applies knowledge of vulnerable populations to the subject consent process and identifies vulnerabilities and applies safeguards for participant protection.* | | **Advanced Level**  Researcher meets the Skilled Level AND can:   1. **Evaluate** a study protocol to identify whether population is properly protected or additional safeguards are needed 2. **Create** strategies to engage vulnerable populations in research studies to allow them to make the best decision 3. **Evaluate** unique situations that affect participation of vulnerable patients 4. **Evaluate** whether vulnerable populations require special considerations from IRBs or regulatory authorities   **Example:** *In a community research study of vulnerable populations, develops strategies that would protect participants during recruitment and retention.* | | |
| 2.6 | Evaluate and apply an understanding of the relevant ethical issues and cultural variation as it applies to the commercial aspects of the clinical research and investigational product development process | | | | | | |
|  | **Fundamental Level**  Researcher can:   1. **Recognize** the cultural variations which exist when conducting multi-regional clinical trials for new investigational product development 2. **Explain** the concept of cultural competency and how it relates to the conduct of clinical research in diverse population groups   **Example:** *Serves as a contributing member of a global medicines development team.* | | **Skilled Level**  Researcher meets the Fundamental Level AND can:   1. **Compare and contrast** the ethical principles guiding clinical research across different global regions (e.g., ICH guidelines vs. FDA regulations, other country regulations) 2. **Examine** the pros and cons of conducting clinical trials in low and middle-income countries and **differentiate** the potential types of exploitation and benefits that populations in these countries may face in the conduct of a global clinical trial   **Example:** *Recommends that clinical studies will only be conducted where the relevant infrastructure exists (e.g. cold chain storage) and in regions where the products will be marketed.* | | **Advanced Level**  Researcher meets the Skilled Level AND can:   1. **Assure** that clinical trials incorporate concepts which recognize varying cultural perspectives and ethical issues across regions 2. **Develop strategies** to select clinical trial sites that appropriately balance the need to provide equal access to potential treatments   **Example:** *Researcher designs a global medicine development program that considers the health needs of potential participants and ensures post trial access to investigational product.* | | |
| 2.7 | Explain why inclusion, exclusion, and other criteria are included in a clinical protocol to assure human subject protection | | | | | | |
|  | **Fundamental Level**  Researcher can:   1. **Recognize** the eligibility criteria for study participants (e.g., that include and exclude subjects) based on factors such as age, gender, the type and stage of a disease, treatment history, and other medical conditions that allows the research team to determine whether the subjects can take part in the study safely 2. **Determine** potential eligibility of study participants for a non-complex study (e.g., registries, survey studies)   **Example:** *Identifies the inclusion and exclusion and eligibility criteria from a set of sample cases for an upcoming clinical study.* | | **Skilled Level**  Researcher meets the Fundamental Level AND can:   1. **Articulate** the necessity for a homogeneous patient population (based on criteria defined in the protocol) and the need for consistency in protocol recruitment 2. **Describe** the implications of deviations from inclusion/exclusion criteria on data quality and study validity and how results can be generalized to the public 3. **Develop** study materials (e.g., guidance documents, recruitment plans) to ensure appropriate application of inclusion/exclusion criteria 4. **Determine** potential eligibility of study participants for complex studies (e.g., biomedical or interventional)   **Example:** *During a study audit, identifies deviations from eligibility guidelines, describes potential consequences, and discusses the required next steps.* | | **Advanced Level**  Researcher meets the Skilled Level AND can:   1. **Develop and edit** eligibility criteria for new protocol development 2. **Explain** the rationale for choosing inclusion and exclusion criteria based on evidence or previous experience   **Example:** *Performs an eligibility risk-assessment and risk mitigation plan for new clinical trials and corrective and preventive action strategies for deviations found during routine site audits.* | | |
| 2.8 | Summarize the principles and methods of distributing and balancing risk and benefit; through selection and management of clinical study subjects | | | | | | |
|  | **Fundamental Level**  Researcher can:   1. **Recognize** the processes (e.g., inclusion/exclusion, study procedures, adverse event identification and documentation, continuation of the study) that appropriately balance risk and benefit   **Example:** *Identifies known and potential clinical risks associated with a clinical protocol and applies ongoing risk assessment activities during study visits with participants.* | | **Skilled Level**  Researcher meets the Fundamental Level AND can:   1. **Implement** the processes (e.g., inclusion/exclusion, study procedures, adverse event identification and documentation, continuation of the study) that appropriately balance risk and benefit   **Example:** *Identifies key risk and benefit components that belong in a Strategic Recruitment and Retention plan or in an Informed consent.* | | **Advanced Level**  Researcher meets the Skilled Level AND can:   1. **Develop** the processes (e.g., inclusion/exclusion, study procedures, adverse event identification and documentation, continuation of the study) that appropriately balance risk and benefit 2. **Illustrate** the risk and benefits principles and methods while **designing** and/or **providing oversight** through the selection and management of clinical study subjects   **Example:** *Independently constructs a protocol, informed consent, and/or recruitment and retention plan that incorporates the principles and methods of distributing and balancing risks and benefits.* | | |
| **DOMAIN 3: Investigational Products Development and Regulation:** *Encompasses knowledge of how investigational products are developed and regulated* | | | | | | | |
| 3.1 | Discuss the historical events that precipitated the development of governmental regulatory processes for investigational products | | | | | | |
|  | **Fundamental Level**  Researcher can:   1. **Identify** the key historical events that took place which influenced the current regulatory environment that exists today (both FDA and internationally)   **Example:** *Understands why the inclusion and exclusion criteria for women of childbearing potential sometimes exists in a clinical study.* | | **Skilled Level**  Researcher meets the Fundamental Level AND can:   1. Demonstrate an understanding of current events that have influenced guidelines and regulatory processes with regards to FDA regulations and guidelines as well as those on a global scale   **Example:** *Locates and describes FDA’s guidance on genomics in clinical research.* | | **Advanced Level**  Researcher meets the Skilled Level AND can:   1. **Predict and/or construct** adaptation plans for the new releases of existing regulations and ICH Guidelines 2. **Support** cross-functional team efforts, provide teaching to internal staff, investigators, and other stakeholders about pending or current guidance or regulations, such as the documentation about training planned for updated ICH E6   **Example:** *Creates a risk-based monitoring plan for a new clinical trial to ensure compliance with FDA regulations and ICH GCPs.* | | |
| 3.2 | Describe the roles and responsibilities of the various institutions participating in the investigational products development process | | | | | | |
|  | **Fundamental Level**  Researcher can:   1. **Identify** differences between responsibilities of investigators, sponsors, CROs and regulatory bodies 2. **Demonstrate** understanding of the role of IRBs in approving protocols, assessing risk, and determining exemptions   **Example:** *Describes the role of an investigator as described in FDA 1572 and the delegation of responsibilities from sponsor to a CRO.* | | **Skilled Level**  Researcher meets the Fundamental Level AND can:   1. **List** specific roles and responsibilities for each of the institutions participating in the investigational products development process, (investigators, sponsors, CROs and regulatory bodies) 2. **Recognize** the scope of responsibilities of monitoring organizations like Research Pharmacy, Data Safety Monitoring Boards   **Example:** *Explains the information required and processes used by the IRB in approving protocols, assessing risk, and determining exemptions.* | | **Advanced Level**  Researcher meets the Skilled Level AND can:   1. **Evaluate** the study protocol to determine the need for collaboration between various institutions/organizations 2. **Define** the roles and responsibilities of the institutions required to complete a research project   **Example:** *Assesses the need and develops a request for proposal for hiring a CRO to conduct monitoring activities for a multicenter trial.* | | |
| 3.3 | Explain the investigational products development process and the activities which integrate commercial realities into the life cycle management of medical products | | | | | | |
|  | **Fundamental Level**  Researcher can:   1. **Understand** concepts, major elements and objectives of investigational products development life cycle management process for investigational products   **Example:** *Has a basic understanding of the drug development and approval process and recognizes the need to obtain approval from the FDA to market the investigational products in US. Maintains site’s IP tracking log at, CRFs, and is familiar with IB or Device Manuals.* | | **Skilled Level**  Researcher meets the Fundamental Level AND can:   1. Interpret and **execute** the concepts, major elements, and objectives of investigational products development life cycle management process for medical products   **Example:** *Uses the FDA website to determine whether a clinical study using investigational products requires an IND or IDE or letter of exemption*. | | **Advanced Level**  Researcher meets the Skilled Level AND can:   1. **Evaluate** an established or create a strategic investigational products development and life cycle management plan 2. **Coordinate** an IP development plan with regulatory authorities 3. **Distinguish** between the regulatory approval processes for drugs, biologics and medical devices   **Example:** *Develops and formulates a request for orphan drug designation for a new investigational product.* | | |
| 3.4 | Summarize the legislative and regulatory framework that supports the development and registration of investigational products and ensures their safety, efficacy and quality | | | | | | |
|  | **Fundamental Level**  Researcher can:   1. **Describe** how to access the appropriate regulatory guidance that applies to the development and registrations of IMPs, and the clinical trials process required to register such products in their geographical location. (US-FDA, Europe-EMeA, UK-MHRA) 2. **Demonstrate** basic knowledge of Human Subjects Protection and ICH GCP guidelines   **Example:** *Accesses the relevant guidance in their country for: Informed Consent, Drug Development and approval, IRBs/ECs, Conflict of interest, Investigator responsibilities, Sponsor responsibilities* | | **Skilled Level**  Researcher meets the Fundamental Level AND can:   1. **Describe and apply** federal (US, EMA, or other) regulatory laws and guidance during the performance of complex clinical research operations. 2. **Interpret** the requirements of ICH GCP, the approved study protocol and sponsor study related SOPs. 3. **Execute** the development or editing of study related SOPs, reports, and / or submission for the relevant regulatory approval of the study.   **Example:** *Describes how regulations and guidance are applied in harmony with ICH GCP requirements, Health Research Authority approvals processes, Research Ethics Committee Approvals and through the comprehensive recording of study related conduct through the maintenance of an investigator site file.* | | **Advanced Level**  Researcher meets the Skilled Level AND can:   1. **Provide oversight and train** others in relation to the relevant authority and associated regulatory frameworks, including how these harmonize with ICH GCP, the approved study protocol, and sponsor study related SOPs to ensure the safety and rights of study participants 2. **Monitor** the progress and assure that conduct of studies at site meets local, national and global regulatory frameworks, and support others to meet such requirements in the conduct of trials   **Example:** *Produces training guides, documentation, and checklists to enable study delivery staff to ensure that the relevant regulatory framework is adhered to in relation to specific studies.* | | |
| 3.5 | Describe the specific processes and phases that must be followed for the regulatory authority to approve the marketing authorization for a medical product | | | | | | |
|  | **Fundamental Level**  Researcher can:   1. **Describe** the specific activities and purposes of preclinical and clinical research and how they contribute to the filing of an IND and an NDA/CTA/BLA 2. **Recognize** how Phase 1-3 data contributes to the filing of an IND and NDA   **Example:** *Participates in the collection of documents necessary for submission of an NDA.* | | **Skilled Level**  Researcher meets the Fundamental Level AND can:   1. Actively **participate** in the implementation of Phase 1-3 clinical trials 2. **Differentiate** between the purposes of the IND, NDA, BLA and each phase of clinical development and the relationship of research questions answered at each phase   **Example:** *Uses the investigator brochure to understand and anticipate what types of potential safety risks might be associated with a clinical trial.* | | **Advanced Level**  Researcher meets the Skilled Level AND can:   1. **Appraise** the potential and resources required for successful implementation of a preclinical or clinical research protocol 2. **Supervise** the development, clinical planning and implementation of a preclinical or clinical research protocol intended to contribute to a regulatory submission (e.g., IND, BLA, NDA) or clinical program   **Example:** *Analyzes data and makes a go/no-go decision after Phase I data are analyzed.* | | |
| 3.6 | Describe the pre- and post- approval safety reporting requirements of regulatory agencies | | | | | | |
|  | **Fundamental Level**  Researcher can:   1. **Identify** the differences between adverse event reporting requirements for studies pre- and post- marketing approval 2. **Understand** the reporting requirements for different types of adverse events   **Example:** *Identifies adverse events that meet the criteria to be labeled ‘serious,’* | | **Skilled Level**  Researcher meets the Fundamental Level AND can:   1. **Assess** the occurrence and coordinate with investigator on classification of adverse events during the conduct of a clinical trial 2. **Complete and submit** adverse event reports, according to appropriate requirements and timeline   **Example:** *Identifies, classifies, and codes an adverse event using source documentation and an appropriate coding dictionary.* | | **Advanced Level**  Researcher meets the Skilled Level AND can:   1. **Identify** and **interpret** safety data (e.g., safety signals or data from surveillance systems) 2. **Mentor** and **teach** others to compare and contrast safety reporting requirements that may differ by region 3. **Comply** with a REMS program.   **Example:** *Serves as the point of contact for both pre- and post-approval safety reporting issues and collaborates with others when responding to questions from regulatory agencies with regards to safety reporting.* | | |
| 3.7 | Appraise the issues generated and the effects of global expansion on the approval and regulation of medical products | | | | | | |
|  | **Fundamental Level**  Researcher can:   1. **Recognize** that different national regulations may affect the medical product approval process   **Example:** *Recognizes that GCP must be honored in multi-site trials, but that other national regulations may differ.* | | **Skilled Level**  Researcher meets the Fundamental Level AND can:   1. Compare regional regulations and how their differences could impact the conduct of trials or the review of medical product approvals   **Example:** *When conducting a study in Japan, applies appropriate strategies to include the correct number of Japanese nationals as part of your study population, as required by the Japanese regulatory agency.* | | **Advanced Level**  Researcher meets the Skilled Level AND can:   1. **Develop** and **implement** strategies for the conduct of multi-regional clinical trials 2. **Develop** and **implement** global strategies that optimize the required review and approval of a marketing application 3. **Analyze** the resources necessary to gain approval for medical products in multiple countries   **Example:** *Knows that a regulatory application in another country may necessitate significantly more resources than a similar application in the US and provides multiple solution alternatives to address barriers to approval of medical products with strategies in alignment with international harmonization efforts (e.g., ICH, EU. WHO).* | | |
| **DOMAIN 4: Clinical Study Operations (Good Clinical Practice):** *Encompasses study management and GCP compliance; safety management (adverse event identification and reporting, post-market surveillance, and pharmacovigilance), and handling of investigational product* | | | | | | | |
| 4.1 | Explain how the design, purpose, and conduct of individual clinical studies fit into the goal of developing a new intervention | | | | | | |
|  | **Fundamental Level**  Researcher can:   1. **Identify** the link between developing a new intervention and the interrelated trial goals and design by reading and comprehending a clinical trial protocol   **Example:** *Identifies the study protocol methods for avoiding selection bias in a clinical study so that the results are considered reliable and valid.* | | **Skilled Level**  Researcher meets the Fundamental Level AND can:   1. **Review** and **comment** on trial protocols to ensure the links between the objective of developing a new intervention and the related trial goal and design is accurate 2. **Provide** input and share ideas, proactively and reactively, on trial design   **Example:** *Reviews and provides substantive editorial comments for a clinical study protocol during its initial development.* | | **Advanced Level**  Researcher meets the Skilled Level AND can:   1. **Design** a clinical trial independently to ensure an accurate link between the goal of developing a new intervention and the trial goal 2. **Train, supervise, and coach** junior trial designers   **Example:** *Independently designs a feasible clinical trial per applicable regulatory requirements, within budget, to provide proof of unbiased safety and efficacy.* | | |
| 4.2 | Describe the roles and responsibilities of the clinical investigation team as defined by Good Clinical Practice Guidelines | | | | | | |
|  | **Fundamental Level**  Researcher can:   1. **Describe** basic principles of GCP 2. **Describe** own role and is aware of roles of others in the site clinical investigation team as set forth by the institution or organization, regulations and GCPs 3. **Understand** the concepts of delegation of authority and scope of practice   **Example:** *Clearly articulates own role responsibilities and describes limits of one’s role in the performance of clinical study activities.* | | **Skilled Level**  Researcher meets the Fundamental Level AND can:   1. **Describe** how GCP principles are incorporated into clinical research 2. **Describe** roles and responsibilities of IRB and sponsors as set forth in federal regulations and GCPs 3. **Performs** role in accordance with GCP guidelines   **Example:** *Accurately identifies and reports situations when clinical investigation team members are not able to fulfill responsibilities and who to contact for support.* | | **Advanced Level**  Researcher meets the Skilled Level AND can:   1. **Apply** GCP Guidelines to the conduct of clinical research 2. **Review and assess** all roles in the clinical investigation team 3. **Supervise** clinical investigation team members 4. **Perform audits** of clinical research performance to ensure compliance with GCPs   **Example:** *Assembles, supervises and manages an appropriate investigational team for multiple clinical research studies* | | |
| 4.3 | Evaluate the design, conduct and documentation of clinical studies as required for compliance with Good Clinical Practice Guidelines | | | | | | |
|  | **Fundamental Level**  Researcher can:   1. Following training, **describe** how the ICH Good Clinical Practice Guidelines are incorporated into the design of a research protocol, the procedures followed during the conduct of a clinical study and the collection of data relating to the study   **Example:** *Describes the concepts contained in the Declaration of Helsinki and how they are incorporated into clinical protocols and implemented during research on human subjects to ensure ethical and quality standards are maintained.* | | **Skilled Level**  Researcher meets the Fundamental Level AND can:   1. Successfully **participate in the implementation** of a clinical research protocol and **assure** that, with minimal supervision, the ICH Good Clinical Practice Guidelines are being followed during the conduct of research procedures and the collection of data   **Example:** *Leads a team that is generating and collecting data in a clinical research protocol in a manner that ensures the conduct, reporting and recording of the clinical study is occurring utilizing internationally accepted guidelines.* | | **Advanced Level**  Researcher meets the Skilled Level AND can:   1. **Ensure** that the operationalization of a clinical research study complies with ICH Clinical Practice Guidelines, 2. Appropriately **resolve** any compliance related issues which arise during the conduct of the clinical study, 3. **Ensure** that the personnel conducting the study are appropriately trained   **Example:** *Assesses and ensures that ICG GCP compliance is maintained throughout the conduct of a clinical research study and when appropriate mentor and train individuals in the ethical and quality concepts required during the conduct of a clinical research study.* | | |
| 4.4 | Compare and contrast the regulations and guidelines of global regulatory bodies relating to the conduct of clinical studies | | | | | | |
|  | **Fundamental Level**  Researcher can:   1. **Describe** the role of global regulatory bodies in the conduct of clinical studies 2. **Identify** the various global regulatory agencies and their respective country-specific regulations 3. **Recognize** the differences in the global regulation of drugs, biologics, and medical devices   **Example:** *Identifies the differences between the regulations and guidelines in the US and Europe for the development and marketing of investigational medicinal products.* | | **Skilled Level**  Researcher meets the Fundamental Level AND can:   1. **Assist** in the identification of country-specific regulations which apply during the conduct of a clinical study 2. **Apply** current processes and procedures for the global regulatory agency application requirements for clinical studies   **Example:** *Applies knowledge of local and global regulations in performing initial feasibility studies for the conduct of global multicenter clinical studies.* | | **Advanced Level**  Researcher meets the Skilled Level AND can:   1. **Create** processes and procedures to determine feasibility for global studies 2. **Determine** and schedule the proper regulatory application requirements and timeframes for study applications 3. **Provide mentoring and educate** others on the global regulatory landscape with respect to the identification of potential clinical sites and the initiation and conduct of clinical studies   **Example:** *Establishes workflows that promote optimal planning for future clinical study applications, data-sharing and clinical sample acquisition for a global multicenter clinical trial.* | | |
| 4.5 | Describe appropriate control, storage and dispensing of investigational product | | | | | | |
|  | **Fundamental Level**  Researcher can:   1. **Understand** that investigational products require specific control, storage and dispensing 2. **Identify** and follow existing Standard Operating Procedures for control, storage, and dispensing of IP   **Example:** *Locates and applies an SOP for the receipt, storage and usage of investigational product for a clinical study at the clinical research site.* | | **Skilled Level**  Researcher meets the Fundamental Level AND can:   1. **Articulate** the specific procedures and elements for control, storage and dispensing of investigational product 2. **Determine** deviations in the process of handling study medication and report /solve the issue   **Example:** *When given a variety of scenarios, implements maintenance of proper environmental storage conditions, security, inventory control, and IP accountability (ordering, receipt, inventory, disposal, transfer) to ensure adequate and safe supplies for clinical study participants.* | | **Advanced Level**  Researcher meets the Skilled Level AND can:   1. **Develop** SOPs that include specific procedures and elements for control, storage and dispensing of investigational product 2. **Develop** CAPAs when issues in the handling of study medication are detected in order to avoid further deviations   **Example:** *Performs audits, generates CAPAs and adjusts SOPs for the management of investigational products according to FDA regulations and GCPs.* | | |
| 4.6 | Differentiate the types of adverse events (AEs) that may occur during clinical studies and explain the identification process and reporting requirement to IRBs/IECs, sponsors and regulatory authorities | | | | | | |
|  | **Fundamental Level**  Researcher can:   1. **Recognize** the differences between the different types of adverse events 2. **Recognize** when an SAE occurs during the conduct of a clinical trial and report it within the appropriate time frame per the regulatory regulations   **Example:** *Applies accurate classification of adverse events from sample cases (AE, SAE, Serious and Unexpected AE, Adverse Drug Reaction, etc.)* | | **Skilled Level**  Researcher meets the Fundamental Level AND can:   1. **Differentiate** the reporting timelines and requirements for an SAE and SUSAR across various international guidelines (e.g., FDA, EMA, ICH, etc.) 2. **Execute** the reporting of an SAE to the appropriate entity (sponsor, regulatory agency, IRB/IEC) based on their respective role (e.g., investigator, CRA, sponsor)   **Example:** *Demonstrates an ability to recognize and report an SAE to the appropriate entity within the appropriate time frame during the conduct of a clinical trial.* | | **Advanced Level**  Researcher meets the Skilled Level AND can:   1. **Critique** the SUSAR reporting requirements across various agencies and entities and **formulate** new recommendations to enhance the harmonization of reporting requirements   **Example:** *Investigates the impact of a lack of harmonization of SUSAR reporting requirements on the timeliness of reporting in a global clinical trial and constructs a new SOP to govern reporting requirements for their organization.* | | |
| 4.7 | Describe how global regulations and guidelines assure human subject protection and privacy during the conduct of clinical studies | | | | | | |
|  | **Fundamental Level**  Researcher can:   1. **Understand** that human research subjects are entitled to protection and privacy and that global regulations are in place to protect research subjects during the conduct of clinical studies 2. **Locate** the specific regulations associated with the protection and privacy of human research subjects   **Example:** *Accurately describes safeguards for human research subject protection and privacy in global, national and local regulations and guidelines.* | **Skilled Level**  Researcher meets the Fundamental Level AND can:   1. **Apply** appropriate protection and privacy safeguards when conducting clinical studies 2. **Report** situations when human research subjects may require protection and privacy 3. **Recogniz**e the existing global regulations and local rules which differ among countries regarding to protect human research subjects and their privacy?   **Example:** *Describes study visit activities, and identifies actions required for subject protection and privacy appropriate for the regulatory body and regulation for different countries (e.g., CFR (FDA, US), EU directive and regulation (EMA, EU), J-GCP (PMDA, Japan), C-GCP (CFDA, China) and guidelines for privacy protection for research participants.* | | | | **Advanced Level**  Researcher meets the Skilled Level AND can:   1. **Create** strategies to protect human research subjects and guard their privacy in clinical studies 2. **Evaluate** whether protection and privacy strategies are appropriate 3. **Develop and implement** a global investigation strategy with global and local regulations to protect human research subjects and their privacy   **Example:** *Plans a new clinical study that includes a comparison of local, national and international health care settings, norms and ethnicities that may impact human subject protection and privacy.* | |
| 4.8 | Describe the role and process of monitoring a clinical study | | | | | | |
|  | **Fundamental Level**  Researcher can:   1. **Recognize and understand** the rationale for clinical monitoring and the appropriate regulations and ICH guidance that applies 2. **Adhere to** the monitoring plan and applicable standard operating procedures 3. With guidance and oversight, **perform** monitoring tasks per the monitoring plan and inform others when confronted with issues not detailed in the monitoring plan   **Example:** *Participates in local QA audits of clinical studies in preparation of a CRO monitoring visit.* | **Skilled Level**  Researcher meets the Fundamental Level AND can:   1. **Employ and implement** the clinical monitoring plan to complete monitoring tasks/activities 2. **Address** complex monitoring issues with minimal supervision or guidance 3. **Provide guidance** to others to **resolve** simple and moderately complex monitoring issues   **Example:** *Applies prospective risk-based approaches to ensure quality data and rapid and accurate responsiveness to clinical monitoring queries.* | | | | **Advanced Level**  Researcher meets the Skilled Level AND can:   1. **Lead** the monitoring effort by mentoring others in the planning and conduct of monitoring site visits 2. **Oversee** **the creation and planning** of study-specific monitoring plans that assure sufficient resources are allocated to ensure timely review of data while maintaining established standards for study participant safety and data integrity   **Example:** *Creates clinical study monitoring plans, provides leadership, mentoring and guidance to ensure all monitoring activities and workflows are in compliance and are ‘audit-ready’* | |
| 4.9 | Describe the role and purpose of clinical study audits | | | | | | |
|  | **Fundamental Level**  Researcher can:   1. **Describe** the steps taken to prepare for an audit/inspection 2. **Name** the entities which have authority to conduct audits 3. **Locate and explain** the federal regulations governing audits and inspections   **Example:** *Assists with preparation for clinical study audits and understands roles of the team during an audit.* | **Skilled Level**  Researcher meets the Fundamental Level AND can:   1. **Distinguish** between scope of audits conducted by sponsors, IRB and regulatory authority 2. **Identify** research components inspected during a clinical study audit 3. **Distinguish** between routing and for-cause audits and inspections   **Example:** *Given a clinical study protocol, classifies and categorizes the specific information and sources of data required by auditors and inspectors.* | | | | **Advanced Level**  Researcher meets the Skilled Level AND can:   1. **Supervise** preparation for an audit/inspection conducted by a sponsor or regulatory authority 2. **Develop** policies and SOPs in response to audit/inspection findings   **Example:** *Given an audit report, creates a comprehensive CAPA plan to respond to audits/inspections, and develop appropriate SOPs.* | |
| 4.10 | Describe the various methods by which safety issues are identified and managed in clinical studies | | | | | | |
|  | **Fundamental Level**  Researcher can:   1. **Understand** that safety is a central issue in clinical trials and that lack of safety oversight can jeopardize participants in numerous ways 2. **Recognize** the tools and processes implemented in a clinical trial to protect participants 3. **Remember** to report suspicious activities or events which might compromise safety   **Example:** *Identifies safety issues, risk mitigation and action plans for diabetic patient who are required to be fasting for a lengthy study visit.* | **Skilled Level**  Researcher meets the Fundamental Level AND can:   1. **Execute** safety reporting within required timelines through appropriate channels 2. **Classify** safety issues and report them to regulatory authorities and IRBs 3. **Implement** international guidelines and requirements across relevant agencies (e.g., FDA, EMA, ICH, etc.) 4. **Relate** safety issues according to monitoring and pharmacovigilance plans   **Example:** *Generates SOPs for the handling of safety hazards in the clinical research site and detecting and reporting adverse events.* | | | | **Advanced Level**  Researcher meets the Skilled Level AND can:   1. **Anticipate** possible safety issues during the clinical study implementation 2. **Institute** measures to minimize risks 3. **Critique and improve** monitoring and pharmacovigilance plans 4. **Recommend and conduct** safety training for study teams   **Example:** *Develops a CAPA plan and staff training for monitoring findings of under-reported adverse events.* | |
| **DOMAIN 5: Study and Site Management:** *Encompasses content required at the site level to run a study (financial and personnel aspects). Includes site and study operations (not encompassing regulatory/GCPs)* | | | | | | | |
| 5.1 | Describe the methods used to determine whether to sponsor, supervise or participate in a clinical study | | | | | | |
|  | **Fundamental Level**  Researcher can:   1. **Demonstrate** a basic understanding of baseline determinants of new study selection process at a research site 2. **Understand** the purpose of pre-site evaluation visits 3. **Participate** in virtual or face-to-face pre-site visits   **Example:** *Given a new potential protocol, understands study-related needs in order to be able to do the study at the site, including availability of a specific study population.* | **Skilled Level**  Researcher meets the Fundamental Level AND can:   1. **Provide** input and guidance in the study selection process, including the ability to assess financial and logistical feasibility of conducting a study at the research site 2. Assist in **organizing and conducting** pre-site visits 3. Assist in **estimating** budgets for a potential study.   **Example:** *Completes a feasibility assessment checklist for a new potential study, including preliminary budget estimates.* | | | | **Advanced Level**  Researcher meets Skilled Level AND can:   1. **Guide** study selection on a program or institutional level 2. **Defend** study selection decision-making, including determination of scientific validity and value; favorable risk/benefit ratio, and operational (logistical and financial) feasibility 3. **Lead** the negotiation, creation of tools, guidance documents, and policies to **guide** the decision-making process in study selection and participation   **Example:** *Creates a study feasibility tool for use throughout department and evaluate assessments to make recommendations.* | |
| 5.2 | Develop and manage the financial, timeline, and personnel resources necessary to conduct a clinical study | | | | | | |
|  | **Fundamental Level**  Researcher can:   1. **Identify** the component parts of a clinical trial budget   **Example:** *Organizes study visits and requisite labs using correct requisition and account numbers for the study and is able to track and reconcile those documents.* | **Skilled Level**  Researcher meets the Fundamental Level AND can:   1. **Critique** and recommend changes to proposed financial budgets, timelines, and amount/type of personnel necessary to conduct a clinical study 2. **Monitor** the progress of a clinical study towards milestones and **identify** trends or risks during study execution. Implements mitigation plans   **Example:** *Analyzes a study budget to ensure all requirements of the protocol are included.* | | | | **Advanced Level**  Researcher meets the Skilled Level AND can:   1. **Develop** the budget, timeline and/or personnel resources to conduct a clinical study; 2. **Identify** trends and **implement** mitigation plans 3. **Manage** personnel that is assigned to the clinical study   **Example:** *Generates amendments to a study budget and milestone timeline to reflect new requirements for an amended protocol and to address unforeseen cost issues for the conduct of a clinical study.* | |
| 5.3 | Describe the management and training approaches to mitigate risk to improve clinical study conduct | | | | | | |
|  | **Fundamental Level**  Researcher can:   1. **Identify** the mechanisms used in a research study that have been put in place to mitigate risk 2. **Understand** how risk assessments are conducted for clinical study operations and patient safety   **Example:** *Articulates potential reasons why a key performance indicator might be compromised (e.g., study participants not completing study visits within the protocol-defined study window) and operations that might ensure lowest risk of occurrence.* | **Skilled Level**  Researcher meets the Fundamental Level AND can:   1. **Identify and understand** the importance of the quality management plan (QMP) and **teach** others about the overall scope of the QMP 2. **Implement** risk mitigation steps as defined in the plan and **develop** a strategy to educate others on its content and application   **Example:** *Analyzes reports and implement defined risk mitigation steps when key performance indicators have been triggered.* | | | | **Advanced Level**  Researcher meets the Skilled Level AND can:   1. **Develop** both generalized and study-specific QMP training programs and delivers these programs to others. 2. **Define** key performance indicators for the clinical studies and **incorporate** them into the study specific QMP. 3. **Interpret** internal QA data on key performance indicators and **strategize** to mitigate risk through a corrective and preventive action (CAPA) plan.   **Example:** *Analyzes and reports quality audit findings, presents them as discussion topics for mitigation strategies during staff meetings and/or incorporates them as part of quality management training programs to ensure staff understand how a QMS applies to a clinical study.* | |
| 5.4 | Develop strategies to manage participant recruitment, retention, compliance and track study activities. | | | | | | |
|  | **Fundamental Level**  Researcher can:   1. **Articulate** expected recruitment and retention rates 2. **Identify** and use tools, strategies, and procedures for implementation and tracking of participant recruitment and retention 3. **Describe** local and international regulatory requirements that impact the use of different recruitment tools   **Example:** *Identifies documents and systems used to track recruitment and retention of participants.* | **Skilled Level**  Researcher meets the Fundamental Level AND can:   1. **Interpret** subject recruitment and retention tracking data to determine if changes are needed 2. **Develop** basic methods for capturing and reporting on recruitment and retention 3. **Apply** local and international regulatory requirements to the use of different recruitment tools   **Example:** *Creates a recruitment plan that addresses the needs of the study population with regards to age, gender, distance, and develops participant fliers for IRB submission that will aid in recruitment.* | | | | **Advanced Level**  Researcher meets the Skilled Level AND can:   1. **Innovate** solutions to recruitment and retention challenges incorporating key ethical considerations. 2. **Propose** different recruitment tools based on regulatory requirements of each region / country   **Example:** *Given a scenario of a study with fledgling recruitment or retention, the researcher creates innovative solutions that are evidence-based, clearly address the specific needs of hard-to-reach/engage populations. The solution includes plans for frequent review of the success of the strategies.* | |
| 5.5 | Identify the legal responsibilities, liabilities and accountabilities that are involved in the conduct of clinical studies | | | | | | |
|  | **Fundamental Level**  Researcher can:   1. **Organize and maintain** study regulatory and grants/contracts documents for regulatory and institutional compliance audits 2. **Understand** purpose of study legal materials including: contract, budgets, indemnification, confidentiality disclosure agreements, conflict of interest reporting and IRB approvals in a compliant study site   **Example:** *When asked by an investigator to obtain samples in the freezer to ship to another investigator for a lab-based research project, researcher at the Fundamental Level knows to seek additional advice to ensure that a materials transfer agreement is in place before making the shipment.* | **Skilled Level**  Researcher meets the Fundamental Level AND can:   1. **Organize** and appropriately **process** contracts, materials transfer agreements, budgets, indemnification agreements, confidentiality agreements and conflict of interest reporting. 2. **Develop** and/or follow SOPs that mitigate legal risks in conducting clinical trials   **Example:** *Reviews an informed consent form to ensure that indemnification language in the Clinical Trial Agreement is in line with indemnification statements in the protocol and informed consent form and institutional policy.* | | | | **Advanced Level**  Researcher meets the Skilled Level AND can:   1. **Monitor** systems and **collaborate** with institutional bodies to ensure compliance with legal and ethical requirements in the conduct of clinical research at the organization. 2. **Develop and critique** risk mitigation strategies, associated action plans and issue resolution 3. **Negotiate** legal contracts (including budgets), confidentiality agreements, and conflict of interest documents   **Example:** *Serves on a conflict of interest board for an institution* | |
| 5.6 | Identify and explain the specific procedural, documentation and oversight requirements of principal investigators, sponsors, CROs and regulatory authorities that relate to the conduct of a clinical study | | | | | | |
|  | **Fundamental Level**  Researcher can:   1. **Identify** the regulations and guidelines that describe the requirements that apply to principal investigators, sponsors, CROs, and regulatory authorities in the conduct of clinical research 2. **Describ**e roles of the site team members, including PI; sponsor, CRO, institution and FDA   **Example:** *Catalogues and files all regulatory documents, including informed consent forms and recruitment materials necessary for an IRB submission.* | **Skilled Level**  Researcher meets the Fundamental Level AND can:   1. **Understand and articulate** applicable regulations and accurately follow established processes in place to ensure compliance 2. **Describe** the various team roles (Sponsor, PI) and their responsibilities in the compliant conduct of clinical research. 3. **Describe** the impact of compliance on the safe and ethical conduct of clinical research studies   **Example:** *Processes an IRB submission for a new clinical trial.* | | | | **Advanced Level**  Researcher meets the Skilled Level AND can:   1. **Apply** advanced understanding of regulations and ability to accurately interpret regulatory guidance and mentor others in the translation of regulations into everyday practice. 2. **Create** strategies, policy and procedures to ensure regulatory compliance at a departmental or institutional level 3. **Organize and manage** regular study-related meetings with study staff and the principal investigators.   **Example:** *Generates a delegation of authority log that clearly delineates staff roles in conducting a study according to levels of responsibility and scope of practice.* | |
| **DOMAIN 6: Data Management and Informatics:** *Encompasses how data are acquired and managed during a clinical trial, including source data, data entry, queries, quality control, and correction and the concept of a locked database* | | | | | | | |
| 6.1 | Describe the role and importance of statistics and informatics in clinical studies | | | | | | |
|  | **Fundamental Level**  Researcher can:   1. **Understand** the basic purpose of statistics and informatics as applied in clinical studies (e.g., randomization, sample size, adverse events, analysis, results)   **Example:** *When reviewing a protocol and case report form, recognizes the data points that are associated with analysis of safety and efficacy endpoints.* | **Skilled Level**  Researcher meets the Fundamental Level AND can:   1. **Perform** randomization activities to ensure accurate designation of new study participants 2. **Describe** the statistical requirements to answer the study question (hypothesis) in a study protocol   **Example:** *Generates descriptive statistics to illustrate enrollment and safety data in a study for a staff meeting presentation.* | | | | **Advanced Level**  Researcher meets the Skilled Level AND can:   1. **Develop** a statistical analysis and data management plan for a clinical study   **Example:** *Develops and annotates a case report form for a clinical trial that will ensure accurate data collection in keeping with the study protocol.* | |
| 6.2 | Describe the origin, flow, and management of data through a clinical study | | | | | | |
|  | **Fundamental Level**  Researcher can:   1. **Describe** the basic concepts of clinical data management. 2. **Identify** the various sources of data that contribute to a clinical study and can distinguish the different industry standards to be used in their handling.   **Example:** *Understands the purpose and scope, as well as the process workflow defined in a data management plan.* | **Skilled Level**  Researcher meets the Fundamental Level AND can:   1. **Apply** all aspects of the clinical data management plan (CDMP) to an active clinical study with regards to the flow of data from the site to the clinical database as well as the flow of data from other sources, for example laboratory electronic uploads, EMR transfers, etc. 2. **Manage** queries and recommend whether the flow and quality of the clinical data meets the standards set in the CDMP.   **Example:** *Performs an analysis of the data flow from various sources (e.g., Esource, third-party sources, etc.) to ensure clean data transfers per predefined specifications.* | | | | **Advanced Level**  Researcher meets the Skilled Level AND can:   1. **Create** the clinical data management plan for a clinical study 2. **Analyze** and **modify** standard operating procedures, when necessary to accommodate the inclusion and implementation of new technology in the data management process or new industry-wide initiatives (e.g. data transparency and clintrials.gov requirements or the MRCT initiatives on data sharing, etc.). 3. **Educate** and **mentor** others concerning their role and responsibility in the conduct and management of clinical data across each aspect of the clinical research enterprise.   **Example:** *Participates at an investigator meeting to review the clinical data management process and the responsibilities each PI and site has in the process.* | |
| 6.3 | Describe best practices and resources required for standardizing data collection, capture, management, analysis, and reporting | | | | | | |
|  | **Fundamental Level**  Researcher can:   1. **Identify and apply** standard and best practices for data management in clinical research. 2. **Identify** documents and resources related standards and best practices associated with the collection, data capture, data management, data analysis, and data reporting in clinical research.   **Example:** *When given standardized scenarios, the researcher identifies a standard or best practice (for data collection, capture, management, analysis, and reporting).* | **Skilled Level**  Researcher meets the Fundamental Level AND can:   1. **Implement** industry, federal and GCP accepted standards and best practices for data management in a clinical study. 2. **Perform** data management activities across clinical studies from creation of protocol specific source documents, collection and entry of data and performing quality audits   **Example:** *Collects and enters data into new electronic data collection forms with timeliness, accuracy and low query rates.* | | | | **Advanced Level**  Researcher meets the Skilled Level AND can:   1. **Develop** a data management plan for a clinical study that includes standardized plans for data collection, data capture, data management, data analysis, and data reporting that use industry-accepted standards or best practices.   **Example:** *Develops an annotated CRF for a specific study according to the data management plan for that study.* | |
| 6.4 | Describe, develop, and implement processes for data quality assurance | | | | | | |
|  | **Fundamental Level**  Researcher can:   1. **Identify and understand** processes that assure data quality. 2. **Recognize** whether individual pieces of data collected in a clinical study are attributable, accurate, complete and verifiable from the source data.   **Example:** *Enters and corrects data from a source document into an electronic data collection form.* | **Skilled Level**  Researcher meets the Fundamental Level AND can:   1. Independently **ensure** compliance with data quality related SOPs 2. **Provide** input and share ideas, pro- and reactively, related to data quality and the related processes.   **Example:** *Suggests a change in an eCRF design to a sponsor to help avoid recurrent queries.* | | | | **Advanced Level**  Researcher meets the Skilled Level AND can:   1. **Create/define** data quality related SOPs or study-specific procedures for the conduct of a clinical trial. 2. **Advise** the data management team on data quality related processes that impact the clinical trial team, ensuring a smooth and constructive collaboration and communication between both. 3. **Train** trial staff on data quality related procedures and provide **oversight** and **support** in cases of doubt or risk for non-compliance.   **Example:** *Generates an eCRF that complies with data quality standards defined by the institution or company.* | |
| **DOMAIN 7: Leadership and Professionalism:** *Encompasses the principles and practice of leadership and professionalism in clinical research* | | | | | | | |
| 7.1 | Describe and apply the principles and practices of leadership, management and mentorship in clinical research. | | | | | | |
|  | **Fundamental Level**  Researcher can:   1. **Display** professionalism in the workplace, in attire, attitude, work-ethic and quality products 2. **Identify** the leadership structure of the organization 3. **Locate, comprehend, and adhere to** the standard operating procedures in the research department 4. **Demonstrate** initiative and team cooperation in performing research duties   **Example:** *Arrives at work on time, articulates information in a succinct and appropriate manner both verbally and in writing, and seeks guidance or directions where he/she has questions.* | **Skilled Level**  Researcher meets the Fundamental Level AND can:   1. **Assist** others with various aspects of study management using effective communication methods and documentation 2. **Train and mentor** Fundamental Level staff 3. **Demonstrate** effective time management and organizational skill when managing multiple research related projects   **Example:** *Plans and conducts a protocol implementation meeting.* | | | | **Advanced Level**  Research Professional meets the Skilled Level AND can:   1. **Serve in leadership roles** in the research department 2. **Train and mentor** new staff members and team members. 3. **Manage multiple** complex study operations 4. **Set strategic planning goals** and objectives for study performance   **Example:** *Manages study teams and develops budgets and assists with contracts for clinical research projects.* | |
| 7.2 | Identify ethical and professional conflicts associated with the conduct of clinical studies and implement procedures for their prevention or management. | | | | | | |
|  | **Fundamental Level**  Researcher can:   1. **Explain** the nature and historical instances of ethical and professional conflicts which occur in the conduct of clinical research 2. **Describe** the procedures which are implemented to prevent ethical conflicts and support risk management strategies   **Example:** *Describes how the concepts within historical documents (e.g., of the Nuremburg Code, the Declaration of Helsinki, the Belmont Report and the CIOMS International Ethical Guidelines for Research Involving Human Subjects) concerning research ethics are integrated into a clinical research protocol.* | **Skilled Level**  Researcher meets the Fundamental Level AND can:   1. **Recognize, implement, and manage** the procedures in a clinical research study which minimize the risks of ethical and professional conflicts 2. **Implement** risk management strategies within their role responsibilities   **Example:** *Organizes and implements the procedures (such as participant recruitment strategies and informed consent) which are included in a clinical research protocol that mitigate ethical and professional risks to clinical trial integrity and contributes to risk management planning for a study team.* | | | | **Advanced Level**  Researcher meets the Skilled Level AND can:   1. **Assess** the risk of ethical and professional conflicts inherent in a clinical study 2. **Develop** strategies and policies to implement and manage risk of ethical and professional conflicts across a project team as well as functional domains   **Example:** *Appraises the potential risks (both ethical and professional) inherent in the conduct of a clinical research study and develops the framework for risk management for a department or project team.* | |
| 7.3 | Identify and apply the professional guidelines and codes of ethics that apply to the conduct of clinical research. | | | | | | |
|  | **Fundamental Level**  Researcher can:   1. **Recognize** the key documents which make up the foundation of the regulations that ensure clinical studies are conducted ethically and in a professional manner 2. **Identify and understand** the meaning of ethical and professional behaviors found in both federal regulations and international guidelines addressing ethical conduct in clinical studies.   **Example:** *Identifies the key regulations and guidelines in FDA and ICH documents that ensure ethical conduct in clinical studies.* | **Skilled Level**  Researcher meets the Fundamental Level AND can:   1. **Apply** professional and ethical regulations and international guidelines in each facet of clinical research 2. **Demonstrate** through actions and documentation of tasks during the conduct of clinical research an understanding of how appropriate procedures and processes assure professional and ethical conduct throughout clinical research   **Example:** *In day-to-day activities and tasks, demonstrates professional behavior and ethical integrity through the applications of all established processes and procedures, regulations, and guidelines.* | | | | **Advanced Level**  Researcher meets the Skilled Level AND can:   1. **Evaluate, and modify** when required, internal policies and procedures to ensure that the organization’s code of ethical conduct is in compliance with local law/regulations and/or international guidelines 2. **Mentor (educate) and provide guidance** to all study team and staff members concerning internal processes and procedures which ensure that all aspects of clinical studies are conducted within the bounds of ethical conduct.   **Example:** *Ensures all local and global regulations and guidelines are reflected in standard operating procedures and processes by adapting any established procedures, processes, or workflows to reflect any new or updated regulations and/or guidelines (e.g. training documentation* | |
| 7.4 | Describe the impact of regional diversity and demonstrate cultural competency in clinical study design and conduct | | | | | | |
|  | **Fundamental Level**  Researcher can:   1. **Describe** why it is important to incorporate strategies that account for regional and cultural diversity in the conduct of clinical research 2. **Classify** examples of potential impact that are related to diversity or cultural competency   **Example:** *Suggests strategies to address diversity and cultural competence for a diverse set of potential participants in a clinical study, including age, ethnicity, race, and gender and religion.* | **Skilled Level**  Researcher meets the Fundamental Level AND can:   1. **Apply** regional/country and cultural considerations during study design and conduct 2. **Incorporate** the appropriate regulatory requirements during the implementation of multi-country trials   **Example:** *Recognizes cultural and diversity issues when developing a research idea into a global clinical study.* | | | | **Advanced Level**  Researcher meets the Skilled Level AND can:   1. **Develop specific strategies** or methods for considering culture and region/country when designing and conducting studies in multiple regions/countries 2. **Validate** that regulatory requirements are incorporated into the study design for multi-country trials   **Example:** *Proposes specific strategies that can be employed in each region/country to ensure cultural and regional appropriateness when initiating a new clinical study.* | |
| **DOMAIN 8: Communications and Teamwork:** *Encompasses all elements of communication within the site and between the site and sponsor, CRO, and regulators. Understanding of teamwork skills necessary for conducting a clinical trial* | | | | | | | |
| 8.1 | Discuss the relationship and appropriate communication between Sponsor, CRO and clinical research site. | | | | | | |
|  | **Fundamental Level**  Researcher can:   1. **Understand and describe** the relationships and appropriate communication channels between regulators, sponsors, CROs and research sites   **Example:** *Demonstrates appropriate written and oral communication between stakeholders in the clinical research operation.* | **Skilled Level**  Researcher meets Fundamental Level AND can:   1. **Apply** appropriate professional communication practices in written and verbal interactions with other parties in order to maintain legal and productive relationships during the conduct of a research study   **Example:** *Develops proactive written and oral communication that addresses team related challenges that could impact study execution so that mutually agreed upon solutions can be developed to address the challenges.* | | | | **Advanced Level**  Researcher meets Skilled Level AND can:   1. **Establish and maintain** productive long-term relationships with all participating parties across the research enterprise to sustain efficient, effective and sustainable clinical trials currently and in the future   **Example:** *Anticipates the needs of all parties participating in the research enterprise and serves as a communication mediator when difficult situations arise that have had previous unsatisfactory results.* | |
| 8.2 | Describe the components of a traditional scientific publication. | | | | | | |
|  | **Fundamental Level**  Researcher can:   1. **Identify** the component parts of a scientific publication and the general purpose of each part 2. **Comprehend** that a traditional scientific publication describes the outcomes of a research study in a structured and ordered format to contribute to generalizable knowledge and evidence-based practice   **Example:** *Reviews and discusses a published study associated with an ongoing clinical study protocol.* | **Skilled Level**  Researcher meets the Fundamental Level AND can:   1. **Describe** the methods for a study that has been published and appreciates the basis for the conclusions made from the results obtained. 2. **Search** the literature using key terms to find articles on specific subjects 3. **Explain** the difference between a primary source and a secondary source when citing the professional literature   **Example:** *Composes an abstract for a publication or professional presentation accurately citing the literature using primary source data (e.g., able to trace a secondary source back to the originating primary source).* | | | | **Advanced Level**  Researcher meets the Skilled Level AND can:   1. **Navigate, appraise and assess** the content of all component parts within a traditional scientific publication and communicate a both detailed understanding to staff 2. **Describe** the relationship of the findings from a clinical study to the relevant human population and current practice context 3. **Write and edit** manuscripts as well as **apply** varying journal citation styles when formatting a manuscript   **Example:** *Given the results of a clinical study, generates and edits a manuscript and/or responds to editorial comments and suggestions in order to develop a final and accepted professional publication.* | |
| 8.3 | Effectively communicate the content and relevance of clinical research findings to colleagues, advocacy groups and the non-scientist community. | | | | | | |
|  | **Fundamental Level**  Researcher can:   1. **Explain** the structure and contents of a scientific publication. 2. **Identify and utilize** reliable sources of information which communicate clinical research findings to the scientific and non-scientific communities   **Example:** *Explains the scientific underpinnings of a clinical trial in terms that can be understood by the non-scientific community.* | **Skilled Level**  Researcher meets the Fundamental Level AND can:   1. **Relate** the content and value of clinical research studies to colleagues and the non-scientific community through professional presentations and other verbal and written means   **Example:** *Writes lay summaries of research studies for a journal club or to potential patient populations.* | | | | **Advanced Level**  Researcher meets the Skilled Level AND can:   1. **Design** reports for scientific and non-scientific communities which interpret and explain clinical trial data and appraise the significance of clinical study reports 2. **Facilitate** the awareness and further understanding of clinical research protocols and their results to colleagues, advocacy groups and the non-scientific community   **Example:** *Communicates outcomes of a clinical research study to sponsors, colleagues and the non-scientific community.* | |
| 8.4 | Describe the importance of team science and methods necessary to work effectively with multidisciplinary and inter-professional research teams. | | | | | | |
|  | **Fundamental Level**  Researcher can:   1. **Describe and understand** the importance of an interdisciplinary team and the values each member can bring to clinical studies 2. **Identify and recognize** each member of the team and their respective roles and responsibilities and understand that communications within a clinical study team is vital to the success of the study   **Example:** *Understands the professional roles and clinical practice domains of all members of the clinical study team.* | **Skilled Level**  Researcher meets the Fundamental Level AND can:   1. **Identify and facilitate** the activities of the key contacts essential to ensuring effective team operations during a clinical study 2. **Demonstrate** an understanding of the cross-functional team in developing a communication plan   **Example:** *Demonstrates the ability to perform the day-to-day operational activities critical to running an effective team (e.g. setting up meetings, developing a communications plan, identification of key contacts both within the team and outside of the team).* | | | | **Advanced Level**  Researcher meets the Skilled Level AND can:   1. **Mentor** others how to work best on a multi-functional clinical study team 2. **Establish** the core infrastructure of the clinical study team and ensure effective and efficient communication and teamwork 3. **Incorporate** multidisciplinary skills into research teams   **Example:** *Creates study teams and establishes an operational workflow to implement study team communication, cross-training, ensures training documentation is maintained, and provides guidance when needed in order for them to optimize their effectiveness.* | |
